# Mali: achieving success along the path to trachoma elimination

**Published:** 2013

**Authors:** Programme National de Lutte contre la Cécité, Helen Keller International, The Carter Center

**Affiliations:** Bamako, Mali.; Bamako, Mali and New York, NY, USA.; Bamako, Mali and Atlanta, GA, USA.

**Programme National de Lutte contre la Cécité**

Bamako, Mali.

**Helen Keller International**

Bamako, Mali and New York, NY, USA.

**The Carter Center**

Bamako, Mali and Atlanta, GA, USA.

Trachoma, the world's leading infectious cause of blindness, affects over 300 million people globally. Caused by the bacterium *Chlamydia trachomatis*, the disease thrives in environments with poor access to water, sanitation, and hygiene. It is spread from one person to another by eye-seeking flies, and by sharing cloths used to wipe the eyes and hands. Repeated or persistent infection can lead to lid scarring and the inward-turning of the eyelid, so that each time a person blinks their eyelashes scrape against the globe of the eye. This incredibly painful condition, known as trichiasis, damages the cornea and eventually leads to blindness.

The World Health Organization has endorsed the implementation of the SAFE Strategy, which is a combination of activities designed to eliminate blinding trachoma. **S** stands for surgery of the upper eyelid to correct trichiasis and preserve sight. **A** stands for the mass distribution of antibiotics (Pfizer-donated Zithromax®, and tetracycline) to clear the eyelid of active infection. **F** stands for facial cleanliness to reduce the presence of infectious ocular and nasal discharge. **E** stands for environmental improvement to improve household access to water and latrines for better sanitation and hygiene. Implemented concurrently and successfully, the four components of the SAFE Strategy provide endemic countries with the tools needed to achieve trachoma elimination.

**Figure:**
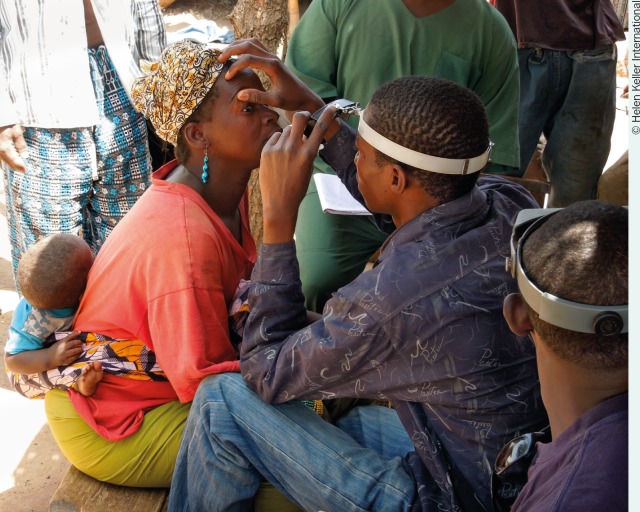
A woman is screened for trichiasis in the district of Sikasso.

Mali, a land-locked country with 16.8 million people in West Africa, has historically been a country with a heavy burden of trachoma. In the late 1990's, the prevalence of active trachoma – also known as follicular trachoma (TF) – was found to range from 23.1% to 46.7% and the prevalence of trichiasis to be 2.5%. This evidence led to the implementation of a trachoma control programme through the National Blindness Prevention Programme (PNLC) in 1998.

Since its inception, the PNLC has made significant progress towards the goal of eliminating trachoma as a cause of blindness by 2015, ahead of the global elimination date of 2020. With support from a multitude of partners, the PNLC has become a leader in trachoma elimination across sub-Saharan Africa. Mali's military coup d’état in March 2012 resulted in the loss of significant donor support to its government, the seizure of the three northern regions (Gao, Kidal, Tombouctou) from the rest of the country, and unprecedented political and social instability. However, the persistence of the PNLC, together with continued financial support from some partners, ensured that their important work continued in all accessible areas during this difficult time.

## Milestones

Major milestones have been reached in the S and A components of the SAFE strategy in Mali. Since 2009, the PNLC has decreased the surgical backlog by almost half with targeted programme planning at the central, regional, and district levels and strategic deployment of human resources (trichiasis surgeons), equipment, and consumables.

**Figure:**
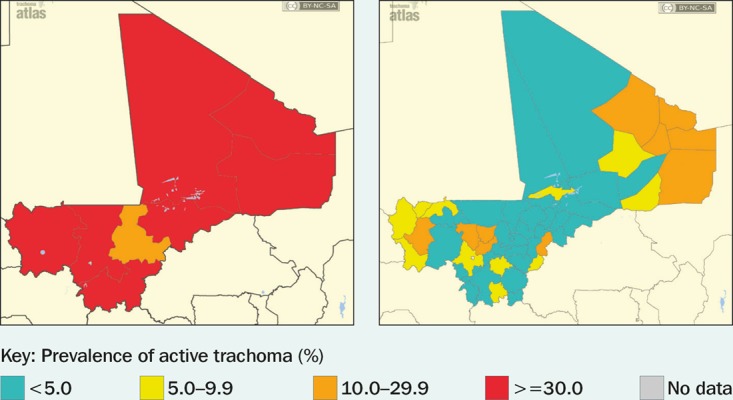
Figures 1 and 2: Maps of Mali depicting the prevalence of active trachoma at the start of the programme (left) and now

The prevalence of active disease has decreased to levels below the threshold recommendation for district-level mass drug administration (MDA), and so the programme has been able to stop this activity in 84 % of the districts where trachoma is present (Figures 1 and 2). This has been due to the high annual rates of coverage with Zithromax® and tetracycline during MDA, strong data collection efforts, and conducting surveys to assess impact.

To address the F and E components, the PNLC conducted several different activities at the same time. These were:

- training for a variety of community groups and leaders (local women's groups, religious/village leaders, and community volunteers) in trachoma prevention
- broadcasting of health messages on community radio stations
- development of a trachoma school health curriculum that is being taught in primary schools
- household latrine construction and community-led total sanitation. Since 2009, PNLC support has assisted in the construction of 53,090 latrines.

## Future plans

The PNLC and partners will continue to build upon the gains made over the past 5 years and support the planning and implementation of SAFE strategy activities. The national programme is refining its surgical planning in order to reach the remaining 27,000 people estimated to need trichiasis surgery, thereby achieving the ‘elimination goal’ of less than one case of trichiasis per 1,000 persons.

Simultaneously, MDA to reduce transmission of trachoma will continue in communities where the prevalence remains high. Surveillance will also continue in areas where MDA has stopped. Social mobilisation and community sensitisation through radios, community volunteers, and women's groups will play a vital role in supporting attitudes and behaviours that help prevent the transmission of disease, strengthen disease knowledge, and decrease the number of people who refuse treatment or surgery. Ongoing latrine construction will continue to provide household access to safe disposal of faeces.

*With thanks to Sanoussi Bamani, Seydou Goita, Yaya Kamissoko, Sadi Moussa, Sidi Coulibaly, Aryc W. Mosher, and Emily Toubali for their contributions to this article*.

